# Probing the allosteric NBD-TMD crosstalk in the ABC transporter MsbA by solid-state NMR

**DOI:** 10.1038/s42003-023-05617-0

**Published:** 2024-01-05

**Authors:** S. Y. Phoebe Novischi, Andrea Karoly-Lakatos, Kerby Chok, Christian Bonifer, Johanna Becker-Baldus, Clemens Glaubitz

**Affiliations:** Institute for Biophysical Chemistry and Center for Biomolecular Magnetic Resonance, Goethe University Frankfurt, Max von Laue Str. 9, 60438 Frankfurt, Germany

**Keywords:** Solid-state NMR, Biophysical chemistry, Membrane proteins

## Abstract

The ABC transporter MsbA plays a critical role in Gram-negative bacteria in the regulation of the outer membrane by translocating core-LPS across the inner membrane. Additionally, a broad substrate specificity for lipophilic drugs has been shown. The allosteric interplay between substrate binding in the transmembrane domains and ATP binding and turnover in the nucleotide-binding domains must be mediated via the NBD/TMD interface. Previous studies suggested the involvement of two intracellular loops called coupling helix 1 and 2 (CH1, CH2). Here, we demonstrate by solid-state NMR spectroscopy that substantial chemical shift changes within both CH1 and CH2 occur upon substrate binding, in the ATP hydrolysis transition state, and upon inhibitor binding. CH2 is domain-swapped within the MsbA structure, and it is noteworthy that substrate binding induces a larger response in CH2 compared to CH1. Our data demonstrate that CH1 and CH2 undergo structural changes as part of the TMD-NBD cross-talk.

## Abstract (web summary)

A solid-state NMR study on the ABC transporter MsbA demonstrates the interplay between transmembrane- and nucleotide-binding domains via both coupling helices.

## Introduction

ATP-binding cassette (ABC) transporters form a membrane protein superfamily stretching across all domains of life. They can be classified as exporters and importers and translocate a wide range of endogenous (e.g., lipids, peptides, vitamins, steroids, and metabolites) and exogenous compounds (e.g., drugs) across plasma membranes. They are responsible for multidrug resistance in bacteria and human cancer cells and were also found to be involved in diseases such as cystic fibrosis, Stargardt disease, retinitis pigmentosa, and others^1–7^.

ABC transporters utilize ATP for transport. They share a similar architecture with two substrate-specific α-helical transmembrane domains (TMDs) and two highly conserved nucleotide-binding domains (NBDs) (for a recent review see ref. ^8^). The NBDs consist of a RecA-type ATP-binding core with four α-helices and 6 β-sheets in complex with an α-helical subdomain (ABCα) and a three-stranded antiparallel β-sheet (ABCβ). Parts of these subdomains form the ATP-binding cassette with the Walker A (RecA), Walker B (RecA), and signature (ABCα) motifs as well as the A- (ABCβ), D- (RecA), Q-loop (RecA) and H-switch (RecA). An additional conserved sequence motif termed X-loop preceding the signature motif has been identified in exporters^9^. In bacterial ABC transporters, these four TMD/NBD domains are either fused into two homo- or heterodimeric polypeptide chains, each with one NBD and one TMD, or they exist as separate subunits.

Cross-talk between NBD and TMD is of central importance for the functional mechanism of ABC transporters since ATP binding and hydrolysis in the NBDs must be allosterically coupled to substrate binding and translocation in the TMDs. The NBD-TMD communication is mediated through long intracellular loops ICL1 and ICL2, which are extensions of transmembrane helices TMH2+3 and TMH4+5, respectively. Both ICLs contain short coupling helices CH1 and CH2, aligned parallel to the membrane, through which they interact with the NBD surface^9–21^. Here, we address how these coupling helices respond to substrate and nucleotide binding in the case of the ABC exporter MsbA.

MsbA (64 kDa) is a homo-dimeric type IV^22^ half-transporter located in the inner membrane of many Gram-negative bacteria such as *E. coli* or *A. baumanii* (see recent review on MsbA^23^). The outer leaflet of the outer membrane of Gram-negative bacteria consists of lipopolysaccharide (LPS), which makes the bacteria inherently resistant to environmental changes. LPS, consisting of lipid A, core oligosaccharide, and O-antigen, reduces the permeability of the outer membrane for many antibiotics, which makes it an important factor in antibiotic resistance^24^. The major function of MsbA is to translocate core-LPS from the inner to the outer leaflet of the inner membrane from where it is transported to the outer membrane by the Lpt system. Core-LPS  is lipid A with covalently attached core sugars^25,26^. This function makes MsbA a major component for maintaining the double membrane architecture in Gram-negative bacteria. As known from multidrug efflux pumps, MsbA also shows broad specificity for a range of amphipathic compounds such as Hoechst 33342^27–32^, which include binding sites other than those for core-LPS^33^. It is therefore considered to be a bacterial homolog of the human P-glycoprotein^34–36^.

The importance of MsbA becomes apparent when considering that Gram-negative bacteria are found amongst the ESKAPE pathogen strains (**E**nterococcus *faecium*, **S**taphylococcus *aureus*, **K**lebsiella *pneumoniae*, **A**cinetobacter *baumannii*, **P**seudomonas *aeruginosa*, and **E**nterobacter species)^37,38^, which contribute to over 40% of the infections in intensive care units^39,40^. Thus, it has been estimated that the mortality rate due to ESKAPE infections will surpass that of cancer in the future^41–45^. MsbA is therefore a potential target for new antibiotics. Recently, two different classes of MsbA inhibitors have been reported: Tetrahydrobenzothiophene (TBT)-based inhibitors block the LPS-binding site and thus transport, resulting in an inward-facing (IF) conformation^46^, whereas quinoline derivatives inhibit LPS translocation by inducing an outward-facing (OF) state that prevents NBD dimerization and thus ATP hydrolysis^47^.

3D structures of MsbA in various membrane mimicking environments have been determined by X-ray crystallography and single particle cryo-electron microscopy covering conformations such as wide-open IF^46,48,49^ and IF^47,49–51^ apo states as well as occluded^51^ and OF^49^ nucleotide-^49,51^, LPS-bound-^47–49,51^ or inhibitor-bound states^46,47^ (see Fig. S1). The wide-open IF conformation with well-separated NBDs was initially debated as artificial due to sample preparation conditions caused by the flat energy landscape of apo-state MsbA but was recently confirmed by EPR spectroscopy in the native membrane directly within *E. coli* cells^52^.

**Fig. 1:**
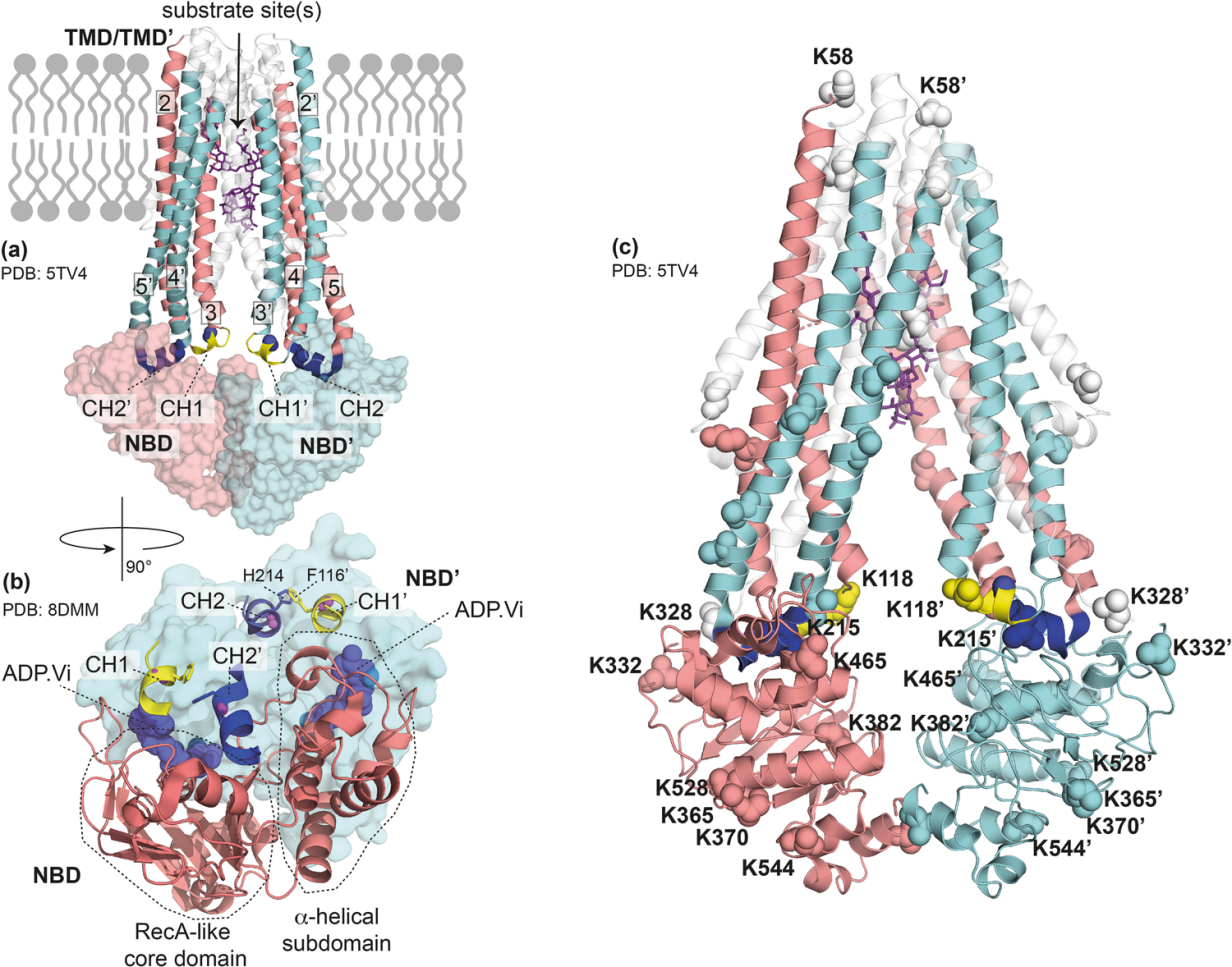
MsbA with substrate- and nucleotide-binding sites, coupling helices, and unique-pair labeling sites. **a** Apo-state MsbA with bound LPS in an inward-facing conformation (PDB 5TV4^51^). CH1 is part of the intracellular loop formed by TMH2 and TMH3. It makes contact with the NBD of the same chain. In contrast, in CH2, the intracellular loop between TMH4 and TMH5 interacts with the opposing NBD’. The ^13^C/^15^N-labeled sites in CH1 (F115-F116) and CH2 (H214-K215) are highlighted as blue spheres. The bound substrate is represented as sticks to schematically show the location of sites where LPS, small ligands, and inhibitors bind. For better visibility, TMHs 1, 1’, 6, and 6’ are only shown as gray transparent. **b** Location of CHs with respect to NBD structural segments based on an ADP•Vi bound MsbA structure (PDB: 8DMM^70^). For simplicity, NBD’ is only indicated as a surface plot. CH1 binds to the NBD surface in the grooves of the RecA-like core domain and comes also in contact with NBD’, while CH2’ is located on the NBD surface in between the RecA-like and the α-helical subdomain. In most structures, F116 of CH1 and H215’ of CH2’ (as well as F116’-H215) point towards each other. **c** Location of Lys residues assigned in NCA spectra of [^13^C,^15^N-K]-MsbA^71^. The residues are highlighted as yellow spheres in the structural cartoon based on the MsbA structure PDB 5TV4^51^.

In MsbA, as in all type IV exporters, two characteristic coupling helices are found. CH1 is part of ICL1, which extends from TMH3+4, and includes residues 113–119 (VSFFDKQ). Analogous, CH2 is found in ICL2 connecting TMH4+5 and is formed by residues 213–221 (GHKEVLIFG) (see Fig. 1a and S1A). CH1 of chain A lies within a groove on the surface of the RecA-like core domain of NBD A. In the NBD-dimerized occluded and OF states, it is also in contact with the surface of the opposite NBD (Fig. S1B). In contrast, CH2 of chain A is domain-swapped which means it lies on the surface of NBD B within a groove at the boundary of the RecA-like core domain and the alpha-helical subdomain. CH2 of chain B then makes the same contact with NBD A (Fig. 1a, b and S1B).

To date, the main evidence for the functional importance of CH1 and CH2 has come from biochemical and genetic data. A full Ala-replacement of CH1 and CH2 in MsbA caused similar effects on nucleotide binding but the CH2 mutant reduced basal ATPase activity much stronger than the CH1 mutant^18^. In the context of extensive EPR studies on MsbA, single-Cys mutations were introduced into CH1 and CH2^53^. Subsequent ATPase assays showed reduced activity for the CH1 mutants but a much stronger reduction and, in some cases, loss of protein stability for CH2 mutants. A related picture emerges from studies on other ABC exporters. For TAP1/TAP2, cysteine cross-linking of CH1 with the X-loop inhibits substrate translocation, whereas cross-linking of CH2 inhibits substrate binding and translocation^16^. In the case of CFTR and P-glycoprotein, NBD mutations at the CH1 and CH2 contact sites also demonstrated their mechanistic importance^54,55^. These results suggest, at least for type IV exporters, that CH2 is functionally particularly important and mediates allosteric coupling between TMD and NBD.

The structural flexibility of the TMD/NBD interface in different conformational states was probed by hydrogen-deuterium exchange mass spectrometry (HDX-MS) experiments for P-glycoprotein and BmrA^56–58^. In the inward-facing (IF) state, CH1, CH2, and their NBD counterparts appear more flexible than in the outward-facing (OF) state, in which, however, CH2 exhibits a greater flexibility than CH1^56,58^. It was also shown that CH1 and CH2 are more rigid in the drug-bound and nucleotide-bound state compared to the post-hydrolysis state^57^.

The available 3D structures of MsbA and other ABC exporters demonstrate how CH1 and CH2 are arranged with respect to both NBDs in distinct conformational states as illustrated in Fig. S1. Based on available P-glycoprotein structures, a ‘ball and socket joint’ model was proposed with CH2 as ‘ball’^59^. However, no clear picture emerged with respect to structural changes within CH1 and CH2 in response to substrate and nucleotide binding.

Nucleotides bind at the canonical binding sites formed at the NBD dimerization interface. MsbA substrates core-LPS and amphipathic drugs as well as quinoline-inhibitors bind to different binding sites within the TMD (Fig, 1a)^33,47,51^. A number of change-in-specificity mutations for small substrates were found in TMH6^60^, which is also part of an inhibitor binding site^47^. The distant nucleotide and substrate binding sites communicate allosterically through the NBD-TMD interface. To obtain experimental evidence for a structural response of CH1 and CH2 as part of this process is therefore important for a mechanistic understanding of NBD-TMD crosstalk.

Here, we address this issue by solid-state NMR (ssNMR) spectroscopy. This NMR approach offers the opportunity to obtain data on the structure and dynamics of membrane proteins within liposomes, which offers a valuable complement to available 3D structures. So far, ssNMR has not been used extensively in the ABC transporter field, but previous studies on MsbA^27,61,62^ have demonstrated its potential to obtain novel mechanistic insight^27,61^ and to probe the conformational space during the catalytic cycle^62^. In the case of BmrA, solid-state NMR was also used to analyze the effect of mutations in the X-loop^63^, which had been previously suggested to play a role for NBD-TMD cross-talk in ABC exporters^9^, and to study the effect of nucleotide binding^64^.

For our approach, we selected Hoechst 33342 from the group of amphipathic MsbA substrates because it has been shown to stimulate ATPase activity and is transported^27–32^. It is also a substrate of other ABC exporters with multidrug specificity^63,65–69^ and has been used by us before for studying substrate-induced effects within MsbA TMH4 and 6^62^. It has also some practical advantages over the endogenous MsbA ligand core-LPS in terms of titratability and also in terms of preparing a clean substrate-free or substrate-bound. Multiple core-LPS species can possibly bind to MsbA and get modulated differently by purification conditions^48,70^.

Here,  we utilize a unique-pair labeling scheme by which CH1 (F115-F116) and CH2 (H214-K215) can be monitored in a highly site-resolved manner (Fig. 1). This approach is combined with a residue-selective Lys-labeling scheme based on known resonance assignments^71^. Substrate-, inhibitor- and nucleotide-dependent chemical shift changes are a sensitive experimental readout for conformational changes. The data described below demonstrates that CH1 and CH2 undergo structural changes as part of the TMD-NBD cross-talk.

## Results

### Labeling scheme selection for CH1 and CH2

For the selective labeling approach used here, suitable residues at sensitive positions within the coupling helices have to be identified. Ideally, these residues should also be part of a unique pair formed with the (i + 1) or (i-1) residues for unambiguous assignment. Furthermore, not all amino acids can be labeled equally well due to isotope scrambling. To select appropriate residues at functionally sensitive sites suitable for labeling, we introduced single-point mutations in CH1 and CH2 and tested their effects using substrate-stimulated ATPase activity assays.

CH1 stretches from residue V113 to G119 (Fig. S1). We have introduced Ala-mutations at positions F115, F116, and D117 in the middle of CH1. All three residues show a high degree of conservation. For all three mutations, a reduction in basal activity is observed, which was especially pronounced for F116A. None of them shows stimulated ATPase activity in the presence of substrate Hoechst-33342 (Fig. S2). The length of CH2 ranges from Gly-213 to Gly-221 (Fig. S2). We probed residues G213, H214, K215, E216, V217 and L218 by Ala-mutations. Of these residues, V217 is especially highly conserved. Here, the strongest effects were observed for H214, K215, and V217 (Fig. S2). A strong reduction in basal ATPase activity and lack of stimulation by substrate is observed for H214A, K215A, and V217A. For G213A, E216A, and K218A, a reduction in basal activity is detected, but their activity can still be stimulated by Hoechst 33342 (Fig. S2). Overall, these data allow the conclusion that mutations in CH2 have a stronger effect compared to CH1, which is in line with previous coupling helix studies on MbsA^18^. Based on these findings and taking known metabolic pathways for isotope scrambling into account^72^, we selected F115-F116 in CH1 for isotope labeling as it is the only Phe–Phe pair in MsbA. For CH2, we choose H214-K215 for further studies, since only two His-Lys pairs occur in the MsbA sequence.

### MsbA apo-state spectra

**Fig. 2:**
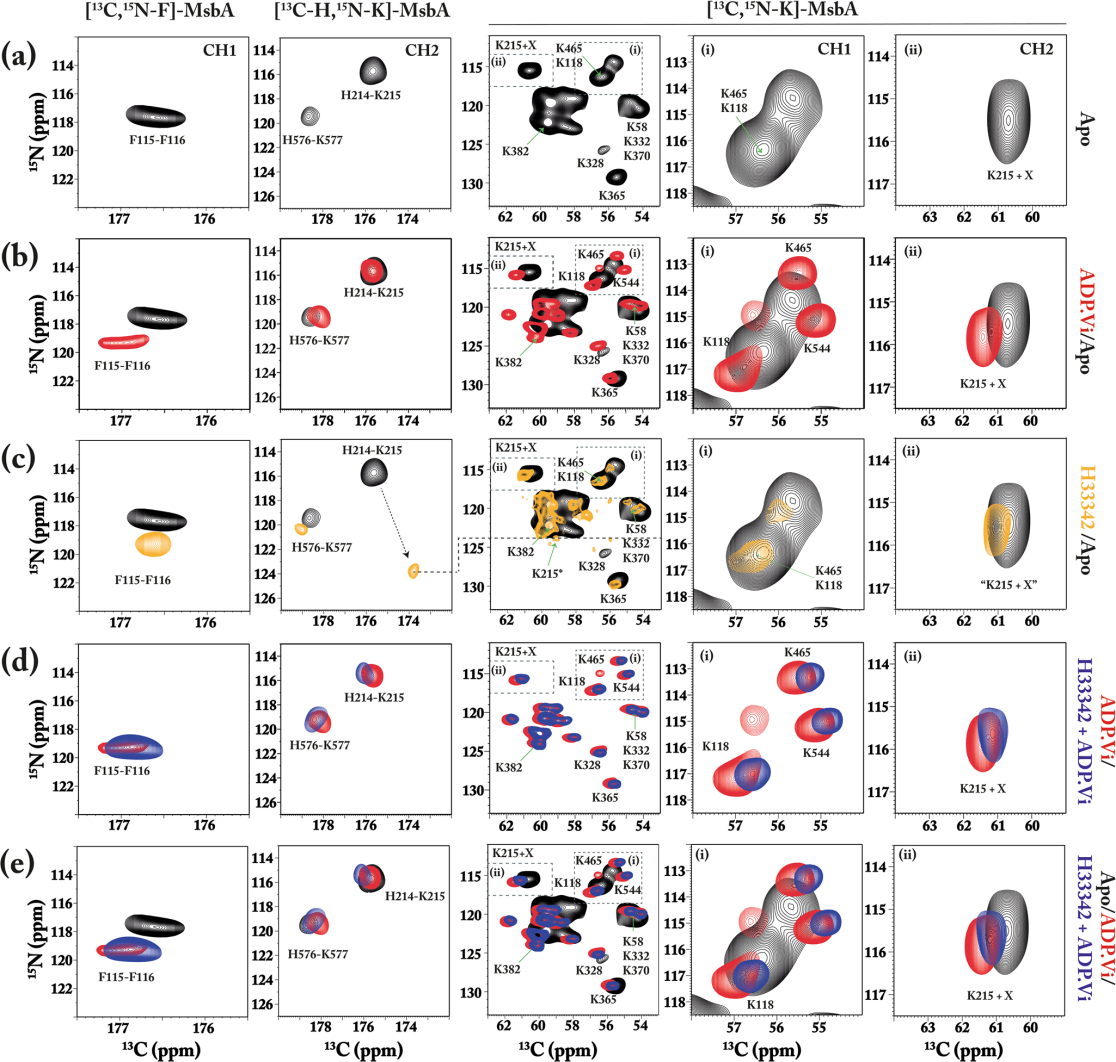
NCO and NCA spectra of residues in CH1 and CH2. NCO spectra of [^13^C,^15^N-F]-MsbA and [^13^C-H,^15^N-K]-MsbA visualize F115-F116 in CH1 and H214-K215 in CH2, respectively (left). In addition, NCA spectra of [^13^C,^15^N-K]-MsbA reveal cross peaks K118 in CH1 and K215 in CH2 (middle, right). **a** NCO and NCA spectra of apo-state MsbA. In MsbA, two H(i)-K(i + 1) pairs occur resulting in two cross-peaks for [^13^C-H,^15^N-K]-MsbA. Some of the Lys in [^13^C,^15^N-K]-MsbA has been assigned in another study^71^. K118 in CH1 overlaps with K465 and K215 with one unassigned Lys (X). The apo state corresponds to an inward-facing conformation. **b** ADP•Vi-trapped MsbA. This state corresponds to an outward-facing conformation. **c** Apo-state MsbA with and without substrate Hoechst 33342. Here, a remarkable shift of the H214-K215 NCO cross peak is observed. The “K215 + X” NCA cross peak (right) does not shift accordingly, which is because only residue X contributes. The K215 NCA cross peak has been tentatively assigned to a spectral intensity matching ^15^N chemical shift (K215*, middle). **d** ADP•Vi-trapped MsbA with and without substrate Hoechst 33342. **e** Superposition of apo-, ADP•Vi- and ADP•Vi+Hoechst 33342 states. The experiments were performed using MsbA in DMPC/DMPA (9:1) with a lipid-to-protein ratio of 75:1. The NCO and NCA spectra were recorded at 600 MHz (270 K, 10 kHz MAS) and 850 MHz (270 K, 14 kHz MAS), respectively.

Two labeled samples were then prepared namely [^13^C,^15^N-F]-MsbA for F115-F116 in CH1 and [^13^C-H,^15^N-K]-MsbA for H214-K215 in CH2. The ^13^C(i-1)-^15^N(i) correlation of the unique pairs can then be visualized in NCO spectra as shown for apo-state MsbA (Fig. 2a, left). While only one cross peak occurs for the [^13^C,^15^N-F]-MsbA sample, two signals can be detected for [^13^C-H,^15^N-K]-MsbA corresponding to both His-Lys pairs H214-K215 and H576-K577. The H214-K215 peak can be unambiguously identified based on the known ^15^N chemical shift of K215 from another ssNMR study on MsbA in which some of the lysines in MsbA have been assigned^71^. We therefore decided to use these data by preparing a third sample, [^13^C,^15^N-K]-MsbA, based on which NCA spectra can be recorded to complement the NCO spectra for CH1 and CH2. An NCA spectrum of apo-state [^13^C,^15^N-K]-MsbA (Fig. 2a, middle) shows several resolved intra-residue lysine cross peaks including K118 in CH1 and K215 in CH2 (Fig. 2a, right, insets i+ii). In the apo state, the signals of K118 and K215 overlap with K465 and with an unassigned lysine (X), respectively. K465 is located prior to the X-loop. Other resolved and assigned residues are K328 (prior to A loop), K365 (between A loop and Walker A motif), and K382 (within Walker A motif). Residues K58, K332, and K370 have been assigned to one overlapping, unresolved cross peak. The location of the assigned lysines is illustrated in Fig. 1c. The NCA spectrum of [^13^C,^15^N-K]-MsbA can serve as a ‘fingerprint’ for the nucleotide-free IF apo state of MsbA.

### Effects of substrate and nucleotide binding

Next, the effect of nucleotide binding on CH1 and CH2 was probed (Fig. 2b). Here, the apo state is compared with the ATP hydrolysis transition state, which is emulated by trapping MsbA with ADP ortho-vanadate (ADP•Vi) resulting in a switch from an IF to an OF conformation. As a result, the CH1 F115-F116 NCO cross peak shifts, while the CH2 NCO correlation of H214-K215 is not much affected (Fig. 2b, left). The NCA spectrum of [^13^C-H,^15^N-K]-MsbA (Fig. 2b, middle) shows many more changes including shifts of the K118 (CH1) and K215 (CH2) cross-peaks (Fig. 2b, right). All three spectra reveal substantial chemical shift increases (>0.5 ppm) for F115 (C’), F116 (N), K118 (N, Ca) in CH1 and K215 (Ca) in CH2 upon trapping (Tab. S1). In addition, the NCO peak of the H576-K577 pair in the NBD shifts (+1.2 ppm for H576-C’). It is noteworthy that the linewidth of the NCA cross peaks of [^13^C,^15^N-K]-MsbA is substantially reduced in the ADP•Vi state.

The effect of substrate binding was then studied by the addition of Hoechst 33342 to the apo sample. In CH1, the F115 nitrogen signal shifts by 1.6 ppm. Even larger changes are observed in CH2 for C’ of H214 and N of K215, which shift by −1.8 and +8.0 ppm, respectively (Fig. 2c, left). In contrast to this observation, the “K215+X” NCA cross peak in the spectrum of [^13^C,^15^N-K]-MsbA does not seem to change (Fig. 2c, right). The reason is that in the apo-state spectrum, K215 and one other Lys residue (X) overlap and contribute to this NCA signal intensity. If only K215 responds to substrate binding but not residue X, then an NCA cross peak will remain at this position. The new K215 NCA cross peak cannot be unambiguously identified but upon inspecting the full NCA spectrum, signal intensity matching the ^15^N chemical shift of K215 as observed in the NCO spectrum can be found (Fig. 2c, middle, “K215*”).

Spectral changes are also observed for lysines in the NBDs, especially for K328 (close to the A-loop) and K365 (between A loop and Walker A motif), which could indicate that substrate binding prepares the protein for ATP uptake. One can speculate that the other observed changes arise from unassigned lysine in the TMD, which could be also influenced by substrate binding.

We then tested how ADP•Vi trapping affects the spectra of Hoechst 33342-bound MsbA. All substrate-affected peaks seem to shift towards their positions in a pure ADP•Vi-trapped state, which is also illustrated in the overlap of all three states (Fig. 2e). Only small changes can be detected between ADP•Vi and ADP•Vi +Hoechst 33342, which are all below 0.5 ppm (Fig. 2d). ADP•Vi trapping in addition to Hoechst 33342 substrate binding seems to set the MsbA protein to an OF state (Fig. 2e).

### Effect of inhibitor binding

Quinolone-based inhibitors bind to MsbA and have been shown to suppress ATP hydrolysis and significantly affect cell growth^47^. We have reproduced the effect of one of these inhibitors, G907, based on cell growth assays using our *E. coli* MsbA expression strain (Fig. S3).

Previous studies have shown that these inhibitors prevent MsbA from going into the OF state by keeping the NBDs separated^47^. However, their binding site is located within the transmembrane domain raising questions about how the TMD-NBD crosstalk mediated by CH1 and CH2 is affected. To address this question, we utilized the MsbA labeling schemes, and the experimental outline described above.

To find the best experimental conditions, the ATPase activity of MsbA in DMPC/DMPA liposomes was probed upon titration of G907. In these liposomes, a surprisingly high stoichiometry of 1:300 was needed to achieve a substantial ATPase reduction (Fig. S4A). Since the binding pocket is within the TMD and accessible from the membrane phase, the reasons could be reduced accessibility and/or reduced G907 membrane penetration in these lipids. The selection of DMPC/DMPA as lipids for reconstitution was primarily driven by previously published studies in which it was shown that MsbA preparations are stable, active, and provide well-resolved NMR spectra^27,61,62,73^. However, it was also shown that the G907 inhibitor affinity was affected by the detergent and lipid environment of MsbA^47^. We therefore reconstituted MsbA into POPE/POPG, which are the main components of the inner *E. coli* membrane, and tested its ATPase activity. Here, we found that a much-reduced stoichiometry of 1:10 is already sufficient to inhibit MsbA (Fig. S4B). Therefore, all further NMR experiments were conducted on POPE/POPG proteoliposomes. A comparison between NCA spectra of [^13^C,^15^N-K]-MsbA reconstituted into DMPC/DMPA and POPE/POPG shows no major chemical shift differences (Fig. S5). Furthermore, POPE/POPG is also closer to the native lipid composition of the inner *E. coli* membrane.

To reach MsbA and its binding site, G907 must be able to cross the outer membrane and penetrate the inner membrane. We therefore analyzed its lipid interactions by ^1^H (NOESY-MAS NMR) spectroscopy. Our data show strong NOEs between G907 protons and protons in the lipid acyl chains of POPE/POPG liposomes. In contrast, cross peaks are weaker in DMPC/DMPA model membranes indicating a lower degree of penetration, which could also explain the differences in the inhibitor efficiency between both lipid compositions (Figs. S6–S8).

**Fig. 3:**
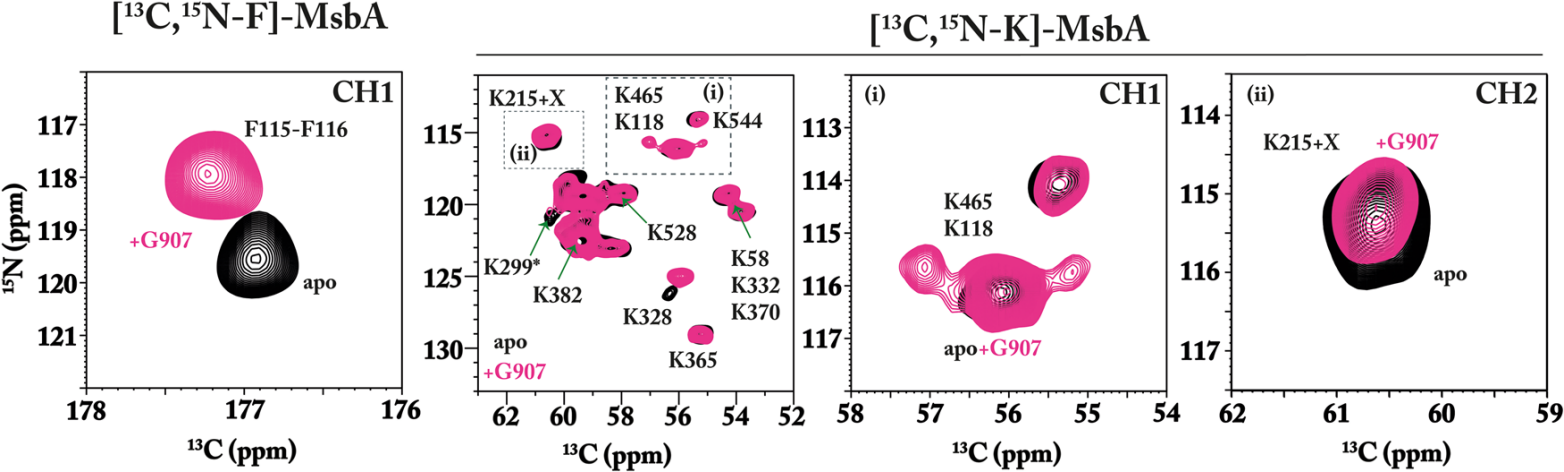
NCO and NCA spectra of residues in CH1 and CH2 upon binding of the allosteric MsbA inhibitor G907. NCO spectrum of [^13^C,^15^N-F]-MsbA (left) reveals a G907-induced shift of the F115-F116 NCO cross peak. The NCA spectrum of [^13^C,^15^N-K]-MsbA (middle, right). The signals of residues (i) K118 in CH1 and (ii) K215 in CH2 overlap with other resonances. Additional intensities occur around K118/K465 upon G907 binding while the K215 cross peak remains unaffected. The experiments were performed using MsbA in POPE/POPG (4:1) with a lipid-to-protein ratio of 75:1. The NCO (CH1) and NCA (13C15N-K + CH2) MAS-NMR spectra were recorded on the 600 MHz (260 K, 10 kHz MAS) and 850 MHz (260 K, 14 kHz MAS), respectively. The apo state is depicted in black. The G907-MsbA is shown in pink.

The effect of G907 onto [^13^C,^15^N-F]-MsbA and [^13^C,^15^N-K]-MsbA was then probed by recording NCO and NCA spectra of MsbA with and without inhibitor present (Fig. 3). In CH1 ([^13^C,^15^N-F]-MsbA), F115(C’) shifts by 0.3 ppm and F116 (N) by −1.6 ppm (Fig. 3, left). Notably, the ^15^N chemical shift in POPE/POPG lipid bilayer is slightly different from the CH1 of MsbA in DMPC/DMPA. Possibly, CH1 is located closely to the interface of the TMD and the lipid bilayer and is more sensitive to these environmental changes. Inspecting the NCA spectrum of [^13^C,^15^N-K]-MsbA reveals also some small G907-induced changes (Fig. 3, right). Additional peaks occur around K118 in CH1, which overlaps with K465 in the apo state (Fig. 3, inset (i)). Peak “K215 + X” shifts slightly by −0.23 ppm (N) (Fig. 3, inset (ii)), which is in contrast to Hoechst 33342 binding, where larger changes were observed (Fig. 2c). This means that K215 is not much affected by G907. In addition, K328 in the NBD shows a 1.5 ppm ^15^N shift. Hence, the effect of G907 seems to affect mainly CH1 and indicates crosstalk. A separate spectrum of [^13^C-H,^15^N-K]-MsbA was not recorded as K215 was already detected in the NCA experiment.

## Discussion

A fundamental question in understanding the functional mechanism of ABC transporters is the interplay between TMDs and NBDs. There are accumulating hints that the coupling helices mediate this crosstalk^9–18^, but to which extent they undergo structural changes is not yet known. Here, we tried to address this question in a highly site-resolved way by creating isotope-labeled C(i)-(N + 1) pairs within both helices so that chemical shift changes can be detected. Although not the full sequence of CH1 and CH2 can be probed in this way, the specific reporter sites provide a very sensitive readout for induced conformational changes during the transport cycle.

We first observed the response of CH1 and CH2 towards nucleotide binding, which induces the transition from an IF- (apo) to an OF-state (see Fig. S1B). The latter was created by trapping the catalytic transition state with ADP•Vi. This conformational switching is reflected by a number of spectral changes in the Lys-NCA spectrum of [^13^C, ^15^N-K]-MsbA (Fig. 2a, b), which involves mainly residues within the NBD (Fig. 1c) and reflects NBD dimerization and nucleotide binding. The observed narrowing of the NCA cross peaks shows that MsbA becomes less flexible upon ADP•Vi trapping. This finding is consistent with HDX studies on MsbA and other ABC exporters^56–58,74^ and also agrees with EPR DEER experiments that showed a narrowing of the broad apo-state distance distributions^52,53,75^.

Both unique pairs within the coupling helices show backbone chemical shift changes that are greater in CH1 (F115/F116) compared to CH2 (H214/K215) (Tab. S1). Chemical shifts of nuclei in the protein backbone mainly reflect the local secondary structure, suggesting that greater structural changes occur within CH1 compared with CH2 during the transition from the IF to the OF state or at least nucleotide binding. Interestingly, ATPase assays (Fig. S2) show a much stronger reduction in activity for H214A and K215A mutants compared to F115A and F116A, which also agrees with published data^18,53^. One might therefore expect a stronger response in CH2, especially when considering its domain-swapped interaction with the opposing NBD, which makes it sensitive to the IF→OF transition. On the other hand, CH2 is located on the surface in between RecA-like and α-helical subdomains, while CH1 lies on top of a groove of the RecA-like domain in close proximity to the bound nucleotide (Fig. 1b and S1B). In the outward-facing state, the NBDs dimerize and CH1 also comes in contact with the surface of the opposite NBD. This could make CH1 more responsive to nucleotide binding and the IF→OF conversion.

Furthermore, the proposed ‘ball and socket joint’ model^59^ does not require secondary structure changes but just rigid body movements of CH2, and alterations in sidechain interactions would not necessarily involve large backbone chemical shift changes. Interestingly, the sidechain of F116 in CH1 of MsbA chain A is oriented towards H214 in CH2 of chain B (Fig. 1b) and it has been suggested that they mediate cross-talk between both coupling helices^18^. They could interact via π-stacking interactions so that both respond to nucleotide binding in a cooperative way, which is compatible with our observation that F116 in CH1 shows large chemical shift changes but mutations in H214 in CH2 have a large impact on the ATPase activity. Both residues are highly conserved in MsbA and F116 is also fully conserved amongst other ABC exporters (Fig. S1). Additional solid-state NMR experiments will be needed in the future to fully describe the interaction between both residues during the ATPase and transport cycle.

For MsbA, it was shown that residues along TM6 are important for binding substrates such as Hoechst 33342^60,62,76^ and that transmembrane helices, in particular TM3 and TM4 which connect to CH1 and CH2 (Fig. 1a), mediate conformational changes between NBD and TMD^30^. Here, upon binding of the MsbA substrate Hoechst 33342, clear chemical shift changes occur in both reporter regions in both coupling helices but the effect for CH2 is especially pronounced with an 8 ppm change for K215-N (Fig. 2c and Tab. S1). Such a large change of backbone chemical shifts is most likely caused by alterations in the local secondary structure and hydrogen bond formations around K215 in CH2, which will also affect the above-mentioned interaction with F116 in CH1. Our biochemical data show that mutations in CH1 abolish the ability to stimulate the ATPase activity of MsbA by Hoechst 33342, which underlines that both coupling helices play an important role in substrate-induced TMD-NBD cross-talk. However, the larger effect on the domain-swapped CH2 indicates that substrate binding induces conformational changes at the TMD-NBD interface, which prepares the protein for ATP binding and subsequent hydrolysis. So far, no nucleotide-free MsbA structure with a bound small molecule ligand (except for inhibitors) has been reported and the LPS-bound forms of MsbA provide no clear conclusion about structural changes within the CHs. However, immobilization of CH1 and CH2 in the peptide exporter TAP1/TAP2 by crosslinking revealed a direct coupling between TMD-NBD crosstalk and substrate binding and translocation^16^. Recent computational studies on P-glycoprotein also suggested a substrate-induced displacement of CH2 which leads to NBD reorientation and pre-dimerization^77^.

Beyond the reporter sites in CH1 and CH2, substrate binding also induces some changes in the Lys-NCA spectrum of [^13^C, ^15^N-K]-MsbA. Some of the peaks shift and the peak intensities change. The cross peaks of lysines in the NBD region adjacent to the A-loop (K328 and K332), Walker A motif (K365 and K370), X-loop (K465), and His-switch (K528 and K544) are affected. These regions normally interact with ATP, suggesting that MsbA is preparing for ATP uptake after substrate binding. But overall, this fingerprint spectrum is not identical to but appears more similar to the IF- (apo) rather than to the OF- (ADP•Vi) state. This observation is compatible with previous suggestions of the formation of a substrate-induced pre-translocation intermediate state^78^, which represents the transition from the IF to the OF-state.

When substrate-bound MsbA is subjected to ADP•Vi trapping, all spectral features change again. The spectra of the coupling helix reporters as well as the NCA Lys-fingerprint spectrum approach the spectral signature obtained for the OF- (ADP•Vi) state. This means that MsbA switches into an OF-state and the substrate-induced changes within CH1 and especially CH2 are reverted. However, the spectra are not identical to the pure ADP•Vi state, which has also been reported in previous ssNMR studies in the effect of substrate binding to TM6^62^. Small spectral changes could also be caused by the non-specific substrate binding or accumulation of Hoechst 33342 within the lipid bilayer.

**Fig. 4:**
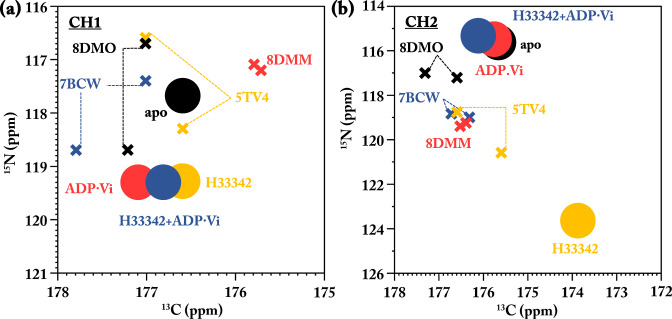
Chemical shift prediction for CH1 and CH2 from selected 3D structures. **a** Comparison between experimental chemical shifts for the F115-F116 unique-pair in CH1 (circles) and predicted NCO cross peaks obtained by ShiftX2^79^ (pH 7.5, 270 K) from the wide-open inward-facing state of MsbA (PDB: 8DMO^70^), the inward-facing conformation (PDB: 5TV4^51^), in the outward-facing state (PDB: 8DMM^70^) and the occluded state (7BCW^80^). In the case of structural asymmetry, two cross-peaks are predicted connected by dotted lines. **b** As in **a** but for the H214-K215 unique pair in CH2.

For an assessment of our findings in the context of known 3D structures, we used the software ShiftX2^79^ to predict the chemical shifts of our unique-pair labels in CH1 and CH2. We selected the wide-open IF state of MsbA (PDB: 8DMO^70^), the IF conformation (PDB: 5TV4^51^), the OF state (PDB: 8DMM^70^), and the occluded state (7BCW^80^) as shown in Fig. S1B. The predicted cross peaks deviate substantially from our experimental observations (Fig. 4). Structural asymmetry leads to the prediction of peak doublets for CH1 in all states and for CH2 in two cases, which are however not observed experimentally in our proteoliposome preparations. We therefore exclude structural asymmetry for CH1 and CH2. Reasons for the deviation between our experimental observation and the structure-based predictions could be limitations in structural resolution and prediction accuracy, crystal packing effects, and the different experimental conditions used for each structure, especially with respect to the membrane mimic and there is no Hoechst 33,342 bound structure so far.

The discussion above focused on the effect of binding of small ligands and nucleotide, which allosterically influence each other,  in turn leading to substrate translocation. The discovery of quinolone-based inhibitors such as G907 with a TMD binding site at TM6 therefore raises the question of how TMD-NBD crosstalk differs, because its mode of action involves disruption of NBD dimerization with subsequent inhibition of ATP hydrolysis^47^.

Here, the binding of G907 resulted in substantial chemical shift changes for F115/F116 in CH1 (Fig. 3 and Tab. S1), while no change was detected for CH2. Interestingly, the X-ray structure of MsbA in complex with G907 in facial amphiphile–3 (FA-3) detergent shows binding-induced propagation of structural changes along TM4 resulting in a larger displacement of CH1 compared to CH2^47^. The proposed inhibition mechanism involves IF-state dependent binding of G907 which prevents transition to the OF state as well as asymmetric NBD-NBD uncoupling. Here, the NCA spectrum of [^13^C, ^15^N-K]-MsbA is similar to the IF- apo-state spectrum but with some specific differences. For example, additional intensities occur around the K118/K465 cross peak and K328 appears shifted. Overall, a general structural asymmetry cannot be concluded from this spectrum, but the additional peak intensities could be an indication. However, since these signals have not been assigned, a definitive statement cannot be made at this point. A recent cryo-EM study of MsbA in complex with a similar inhibitor G247 in nanodiscs proposed a symmetric NBD uncoupling but no specific conclusion on the coupling helices has been derived^46^. In summary, our data show that G907 binding in the TMDs triggers signaling into the NBDs involving at least CH1 and stabilizing an IF state.

**Fig. 5:**
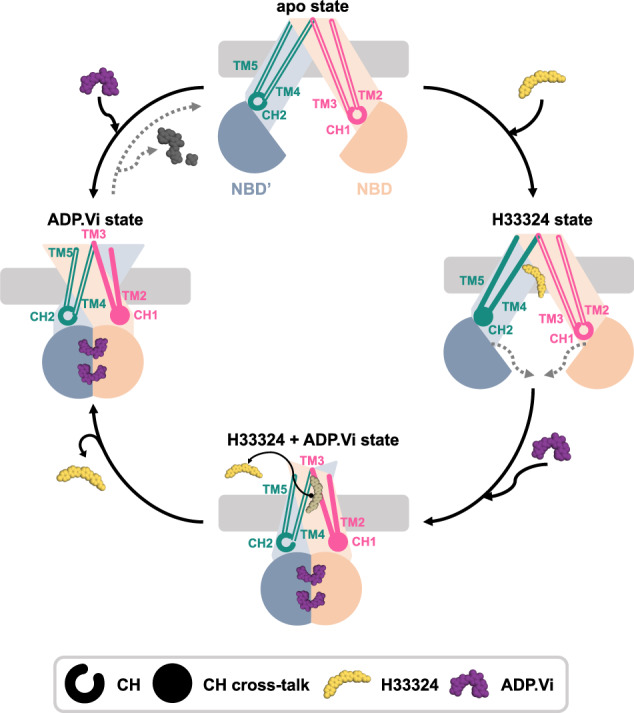
The response of CH1 and CH2 upon nucleotide and substrate binding. In the apo state, CH1 is solely interacting with the NBD of its own chain and CH2 with the other NBD’. Upon binding of Hoechst 33342, MsbA moves to an occluded conformation, and a large chemical shift change is observed within the domain-swapped CH2 (Hoechst 33342 state). It triggers conformational changes within the NBD which supports nucleotide binding. Trapping MsbA in an ATP hydrolysis transition state by ADP•Vi (ADP•Vi state), a conformational change from the inward- to the outward-facing conformation occurs. Here, a chemical shift change in CH1 is observed. ADP•Vi trapping of the Hoechst 33342 state converges towards the ADP•Vi state.

The study presented here provides direct evidence for structural changes within the coupling helices of type IV ABC transporters during the transition from the IF to the OF state and during substrate and inhibitor binding. Our findings are summarized in Fig. 5. The data show that ADP•Vi binding and the IF→OF transition cause at the NBD-TMD interface a stronger response in CH1 while substrate binding has a stronger effect in CH2. It is noteworthy that the latter is based on a domain-swapped interaction with the NBD. Both cases are caused by stimuli with different vectoriality, namely nucleotide binding to the NBD and substrate binding to the TMD, which might then involve different pathways for NBD→TMD and TMD→NBD crosstalk. Our data also demonstrate that CH-mediated crosstalk plays a role in the mechanism of an allosteric MsbA inhibitor, which binds in the TMD but prevents ATP hydrolysis in the NBD. The observed spectral signatures are different compared to the substrate-bound state, which indicates a different interaction pathway. Our study provides selective data, which is highly complementary to the available 3D structures. Future solid-state NMR experiments will address the potential interaction between CH1 and CH2 and connect NMR data and 3D structures via computational approaches.

## Methods

### Protein expression and purification

Wild-type MsbA was expressed and purified as described previously^27,61,62,73^. Using a pET-19b vector containing an N-terminal His10-tag connected via an 11 amino acid peptide linker, the wild-type MsbA gene was cloned and transformed into *E. coli* C43(DE3) cells for protein expression. To increase the yield of isotope-labeled MsbA MAS-NMR samples, expressions were carried out in enriched M9 minimal media (M9^+^).

For coupling, helix 1 [^13^C,^15^N-F]-MsbA 103 mg L^−1 13^C,^15^N-phenylalaline (Cortecnet), and 800 mg L^−1^ 4-hydroxy phenyl pyruvic acid (Sigma Aldrich) were supplemented to 1 g L^−1 14^NH_4_Cl and 2 g L^−1 12^C-glucose M9^+^ media. In the case of coupling helix 2 [^13^C-H,^15^N-K]-MsbA 100 mg L^−1 13^C-histidine or ^13^C,^15^N-histidine (Cortecnet) and 420 mg L^−1 15^N-Lysine (Cortecnet) added to the M9^+^ media. Lastly, the [^13^C,^15^N-K]-MsbA was expressed using 420 mg L^−1 13^C,^15^N-Lysine. To enrich the M9^+^ media for overexpression, natural abundance 500 mg L^−1^ alanine, 400 mg L^−1^ arginine, 550 mg L^−1^ glycine, 230 mg L^−1^ isoleucine, 230 mg L^−1^ leucine, 250 mg L^−1^ methionine, 2010 mg L^−1^ serine, 230 mg L^−1^ threonine, 230 mg L^−1^ valine, 400 mg L^−1^ aspartic acid, 50 mg L^−1^ cystine, 417 mg L^−1^ glutamine, 650 mg L^−1^ glutamic acid, 100 mg L^−1^ proline, 50 mg L^−1^ tryptophan, 100 mg L^−1^, and 170 mg L^−1^ tyrosine were supplemented.

In 1 L of Luria Broth media (25 gL^−1^) 10 mL of 16–18 h at 37 °C preculture was inoculated to initiate protein expression (37 °C). Upon an OD_600nm_ of 0.5–0.6, the cells were washed and transferred to 600 mL of enriched M9^+^ media. Prior to 1 mM isopropyl-β-D-thiogalactopyranoside (IPTG) induction (20 °C) of 16–18 h (260 r.p.m.), the cells were allowed to adapt to the media for 1 h at 37 °C (220 r.p.m.).

Membranes were yielded from harvested and lysed cells (10 mM Tris, 250 mM Sucrose, 150 mM NaCl, 2.5 mM MgSO_4_, 0.5 mM protease inhibitor, 16 mM 1,4-dithiothreitol, and DNase, pH 7.5), passing a French press 3–5 times (I&L Biosystems high-pressure cell disrupter) under a pressure of 1.7–1.9 kbar. Cell debris was removed (4.500 × *g*, rotor F0850, 15 min) prior to ultracentrifugation (223.000 × *g*, rotor 70 Ti, 1 h), resulting in the final membrane fraction.

MsbA was further purified by membrane solubilization (50 mM HEPES, 300 mM NaCl, 5 mM MgCl_2_,10% Glycerol, 1.25% n-Dodecyl-β-D-maltoside (DDM, AppliChem), and 10 mM imidazole pH 7.5, 4 °C, 17 h, 223.000 × *g*, rotor 70 Ti, ultracentrifugation 1 h), followed by His-tag/Ni-NTA purification of the supernatant, yielding 10–15 mg L^–1^ M9 media or 30–35 mg L^–1^ M9^+^ media in HEPES with 0.015% DDM (50 mM HEPES, 300 mM NaCl, 5 mM MgCl_2_ and 10% Glycerol, 0.015% DDM, and 400 mM imidazole).

### Reconstitution in DMPC/DMPA lipids and ADP ortho-vanadate and Hoechst 33342 trapping

Reconstitution of MsbA was performed as in previous studies^27,61,62,73^. Nine mols of 1,2-dimyristoyl-sn-glycero-3-phosphocholine (DMPC, Avanti Lipids) were mixed with one mol of 1,2-dimyristoyl-sn-glycero-3-phosphate (DMPA, Avanti Lipids) in CHCl_3_/CH_3_OH (2:1) solvent and dried under nitrogen gas flow. Accordingly, liposomes were prepared in buffer (50 mM HEPES and 50 mM NaCl, pH 7.5) and extruded >10 times through membranes (100 nm) to form uniform vesicles. Upon detergent destabilization (3 mM Triton X-100, Sigma Aldrich), MsbA was reconstituted in the liposomes with a final lipid-to-protein (LPR) ratio of 75:1 mol/mol. The protein/liposome mixture was allowed to equilibrate for 30 min at room temperature before the removal of detergent (80 mg L^−1^ biobeads, overnight, 4 °C). The reconstituted sample was washed and collected by ultracentrifugation (28,000 r.p.m., rotor Ti70, 20 min). The ATPase activity was comparable to previously reconstituted MsbA in liposomes^73^. Trapping of Hoechst 33342 (Sigma Aldrich) and ADP ortho-vandate in MsbA was achieved as previously described. The trapping of Hoechst 33342 + ADP ortho-vanadate was done by first incubating MsbA with Hoechst 33342 and then with ADP ortho-vanadate. The protein mixture containing 50 mM HEPES, 10 mM ATP, 10 mM MgCl_2,_ and 3 mM ortho-vanadate solution was subjected to freeze-thaw cycles to improve the MsbA accessibility in the proteoliposome sample at 37 °C for 20 min. Subsequently, the sample was washed with 20 mM HEPES to remove excess reagents, pelleted, and packed into a 3.2 mm or 4 mm MAS rotor. Successful trapping was confirmed by ^31^P-CP MAS-NMR^27^ (see exemplary spectrum in Fig. S9).

### ATPase activity assay

MsbA (0.015% DDM) is in an assay buffer (*50* *mm HEPES, 50* *mm NaCl, 10* *mm MgCl*_*2*_)^73,81^. The release of inorganic phosphate was followed by titration of ATP up to 5 mM at OD_850_. The reaction is stopped after 20 minutes by using 12% w/v sodium dodecyl sulfate (SDS) and colored in two consecutive steps. Firstly, with a 1:1 mixture of 12% w/v ascorbic acid and 2% w/v ammonium molybdate in 1 M HCl. Finally, after 5 min use a mixture of 2% w/v sodium citrate, 2% w/v sodium meta-arsenite, and 2% v/v acetic acid with an incubation time of 20 min. Experiments were repeated on 3–5 distinct samples (Figs. S2—S4).

### Reconstitution in POPE/POPG lipids and G907 trapping

Reconstitution of MsbA in 1-palmitoyl-2-oleoyl-sn-glycero-3-phosphoethanolamine (POPE, Avanti Lipids) and 1-Palmitoyl-2-Oleoyl-sn-Glycero-3-Phosphoglycerol (POPG, Avanti Lipids) was done similarly as for MsbA in DMPC/DMPA. POPE/POPG (4:1) was dissolved in CHCl_3_ and dried under nitrogen gas flow. Subsequently, liposomes were prepared in buffer (50 mM HEPES and 50 mM NaCl, pH 7.5) and extruded >10 times through membranes (100 nm) to form uniform vesicles. Upon detergent destabilization (3 mM Triton X-100), MsbA was reconstituted in the liposomes with a final LPR ratio of 75:1 mol/mol. The protein/liposome mixture was allowed to equilibrate for 30 min at room temperature before the removal of detergent (80 mg L^−1^ biobeads, overnight, 4 °C). The reconstituted sample was washed and collected by ultracentrifugation (28,000 r.p.m., rotor Ti70, 20 minutes). MsbA was incubated with the G907 (MedChemExpress) inhibitor (10 mol/mol MsbA) for 1 h at room temperature. The sample was pelleted and packed into a 3.2 mm or 4 mm MAS rotor for further measurements. The inhibitor/protein ratio was determined by the ATPase activity using G907 kindly provided by Genentech (Fig. S4).

### G907 in POPE/POPG sample preparation

The preparation of G907 (MedChemExpress) in POPE/POPG was done as described before for other drug-lipid interaction studies^82^. A total of 10 mg POPE and POPG was dissolved in CHCl_3_ and G907 was added to the mixture to yield a drug/lipid ratio of 1:5 and dried under nitrogen gas flow. Multilamellar vesicles were then prepared by hydrating each sample with approximately 10 μL of D_2_O ( > 97%). The sample was then freeze-thawed 10 times in liquid nitrogen and a 30-degree Celsius water bath. Finally, the gel-like sample was transferred into a 4 mm MAS rotor.

### Solid-state NMR experiments

The NCO and NCA spectra were recorded using Bruker a 600 MHz AVANCE NEO or Bruker 850 MHz AVANCE III NMR spectrometer equipped with Bruker 3.2 mm efree triple resonance MAS probes.

All NCO spectra were acquired with 20-25 mg MsbA reconstituted into DMPC/DMPA. For the NCA experiments, 10–15 mg of MsbA reconstituted into POPE/POPG or DMPC/DMPA was used. The nominal probe temperature was set to 270 K and a MAS rate for 14 kHz (at 850 MHz) and 11 kHz (at 600 MHz) was used. ^13^C-^13^C PDSD spectra of all three MsbA samples were recorded to probe for isotope scrambling (Fig. S10). ^13^C and ^15^N chemical shift referencing indirectly to DSS using the C’-resonance of Alanine at 179.85 ppm.

For all experiments, standard settings for cross-polarization (CP) and decoupling were applied. ^1^H 90° pulse had a duration of 3 µs with a chosen CP contact time between 0.5 and 1 ms and high-power proton decoupling of 70–100 kHz was applied using SPINAL64 during evolution and acquisition. 90°-pulses are set to 4.5 and 6.0 µs for ^13^C and ^15^N, respectively. NCO spectra were recorded with 5120 scans (30 increments) using a spectral width of 2 kHz in ω1. NCA spectra were recorded with 3720 scans (50 increments) using a spectral width of 2 kHz in ω1. Gd^3+^-DOTA doping (3 mM) of MsbA reduced the recycle delay from 3 to 1s. All spectra were processed with TOPSPIN 4.1 using an exponential window function applied to 1D spectra and a Gaussian in the direct and cosine-shifted sine bell function in the indirect dimension of the 2D spectra.

The two-dimensional ^1^H MAS-NOESY spectra (Figs. S7 and S8) were recorded using Bruker 600 MHz AVANCE NEO with a 4 mm double resonance probe. A total of 256 data points were collected in the indirect dimension, with 32 scans and a recycle delay of 2s. All spectra were referenced with respect to HDO at 4.8 ppm or sodium trimethylsilylpropanesulfonate (DSS) at *0.00* *ppm. NOESY spectra were obtained with mixing times between 50 and 400* *ms*.

### Statistics and reproducibility

All biochemical experiments were performed at least three times. All NMR samples were freshly prepared followed by immediate recording of NMR spectra within 2–3 days. All spectra were reproduced on different samples. A sufficiently large amount of sample was used to fill a MAS sample rotor. The NMR spectra were recorded with a large number of scans to ensure a good signal-to-noise ratio. The spectra represent an average over a large number of protein molecules (10^17^) in the sample.

### Reporting summary

Further information on research design is available in the Nature Portfolio Reporting Summary linked to this article.

### Supplementary information

Supporting Information

Description of Additional Supplementary Files

Supplementary Data 1

Supplementary Data 2

Supplementary Data 3

Supplementary Data 4

Reporting Summary

## Data Availability

The paper and the Supplementary Materials provide all the data needed to evaluate the conclusions. All raw data are available upon request. Numerical data for Fig. 4, S2, S3, and S4 are provided as Supplementary Data 1 – 4, respectively.

## Code availability

This work does not contain specialized source codes.
