# Supplementary material for: Probing the allosteric NBD-TMD crosstalk in the ABC transporter MsbA by solid-state NMR
Source: Commun Biol. 2024 Jan 5;7:43. doi: 10.1038/s42003-023-05617-0 (PMC10770068; doi:10.1038/s42003-023-05617-0)
Supplement: Supplementary file 1 — Supporting Information [file 42003_2023_5617_MOESM1_ESM.pdf]

Supplementary information

Probing the allosteric NBD-TMD crosstalk in the ABC Transporter MsbA by solid-state NMR

S. Y. Phoebe Novischi[1], Andrea Karoly-Lakatos[1], Kerby Chok[1], Christian Bonifer[1], Johanna Becker-Baldus[1] and Clemens Glaubitz[1,*]

([1]) Institute for Biophysical Chemistry and Center for Biomolecular Magnetic Resonance, Goethe University Frankfurt, Max von Laue Str. 9, 60438 Frankfurt, Germany

(*) Email: glaubitz@em.uni-frankfurt.de

(A) MsbA Coupling Helices

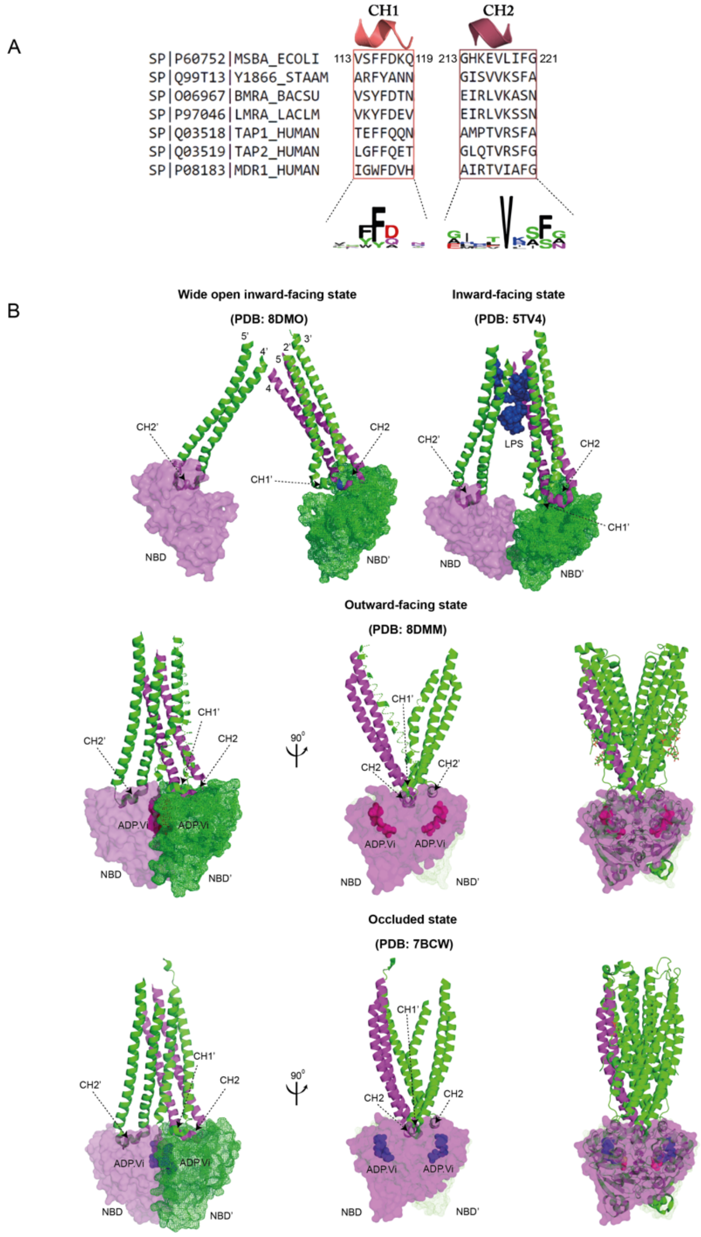

Figure S1: Coupling helix sequences and structural arrangement. **(A)** Sequence alignment of the coupling helices of MsbA and related homo-dimeric exporters Sav1855, BmrA, LmrA, MDR1 as well as hetero dimeric TAP1/2. The degree of conservation is illustrated by WebLogo (https://weblogo.berkeley.edu). **(B)** Arrangement of coupling helices in the wide-open inward-facing state of MsbA (PDB: 8DMO [1]), in the inward-facing conformation (PDB: 5TV4 [2]), in the outward-facing state (PDB: 8DMM [1]) and the occluded state (7BCW [3]). For simplification, all transmembrane helices have been omitted except for 4,5 of one protomer and 2', 3', 4', and 5' of the other. For the outward-facing and occluded states, also a plot with all TMHs is shown (right).

(B) Coupling Helix Mutations

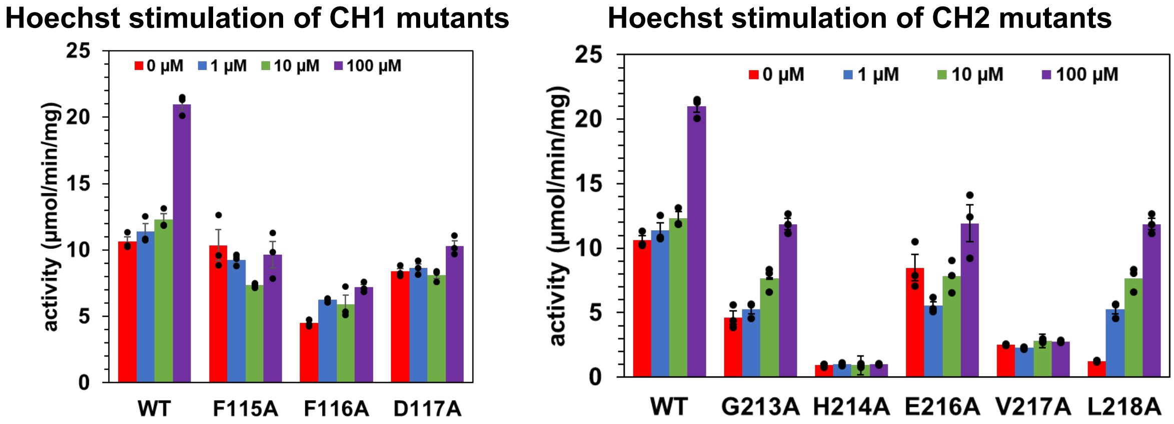

Figure S2: Stimulated ATPase activities of MsbA and coupling helix mutants in DDM. The ATPase activity was stimulated by Hoechst 33342 and determined as described in the Materials and Methods section. The activity reported here corresponds to [ATP] = 1.5 mM (n=3 of distinct samples with mean±SEM). The stimulated ATPase activity is strongly affected by mutations in both coupling helices. Positions F115, F116, and H214, K215 have then been selected for isotope labeling to serve as reporters within the coupling helices.

The MsbA ATPase activity was determined based on previous studies using MsbA proteoliposomes incubated with Hoechst in assay buffer (*50 mm HEPES, 50 mm NaCl, 5 mM ATP, 10 mm $MgCl_2$*).[4,5] The release of inorganic phosphate was detected at a fixed ATP concentration of 5 mM at OD_{850}. The reaction was stopped by using 12% w/v sodium dodecyl sulfate (SDS) and colored in two consecutive steps, firstly, with a 1:1 mixture of 12% w/v ascorbic acid and 2% w/v ammonium molybdate in 1 M HCl and finally, using a mixture of 2% w/v sodium citrate, 2% w/v sodium meta-arsenite, and 2% v/v acetic acid.

(C) Cell growth assay in the presence of MsbA inhibitor G907

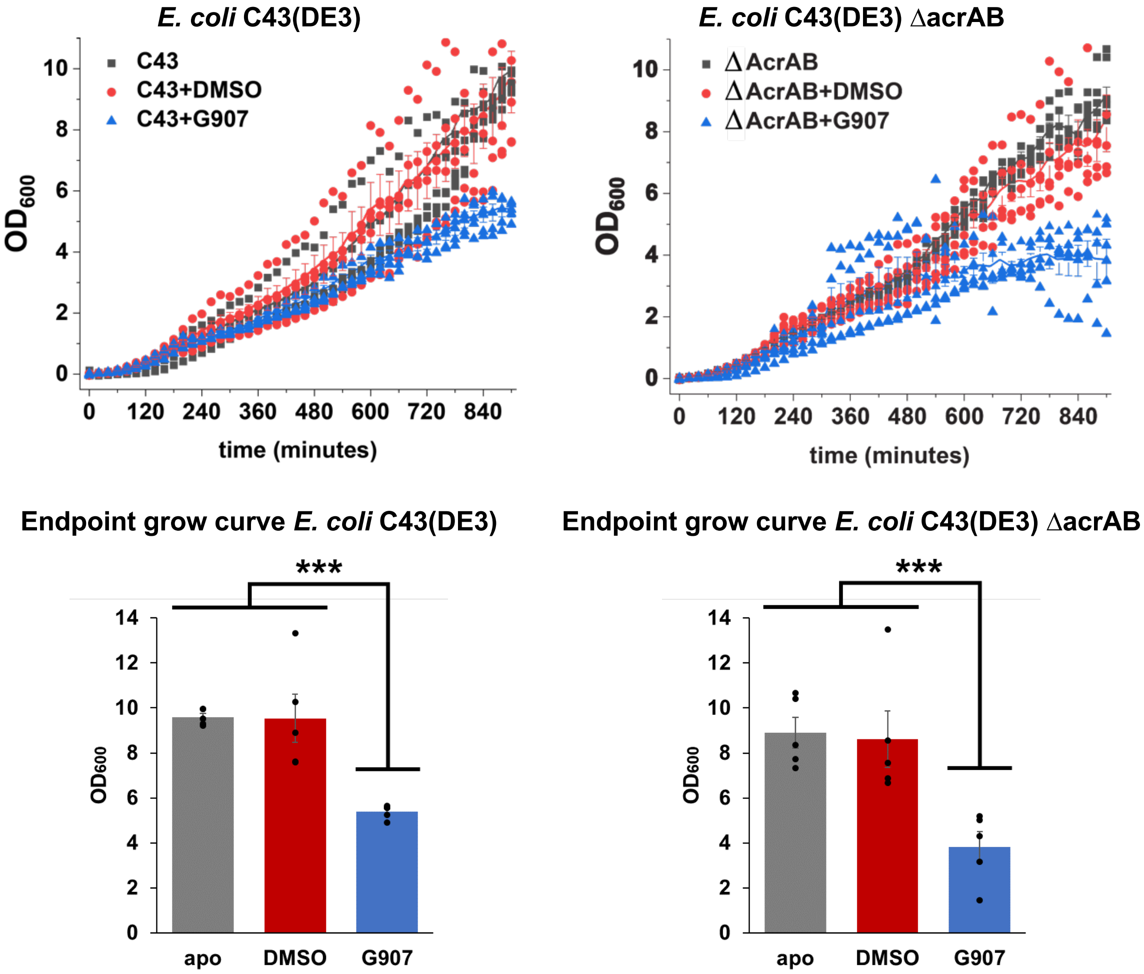

Figure S3: The effect of G907 on the growth of *E. coli* cultures. The MsbA gene was cloned and transformed into *E. coli* C43(DE3) and *E. coli* C43(DE3) ΔacrAB cells. The cells were grown in a preculture overnight (16 h, 37 °C, 220 r.p.m.), washed, and diluted to OD_{600} of 0.01 in 150 µL volume in a sterile microtiter plate in Luria Broth (25 g/L) and ampicillin (100 µg/mL). The growth of the cells was followed over a 12-hour timespan (20-minute interval) using BMG LABTECH CLARIOstar at 37 °C. After steady growth of 240 minutes, the G907 concentration in the culture volume was adjusted to the (minimum inhibitory concentration (MIC)[6,7] of 6 µg/mL by adding a G907 from a DMSO stock solution. The same amount of DMSO was added to the control cells. Growth was followed for the remaining 720 minutes. The matrix data was then processed in Microsoft Excel and transformed into growth curves using OriginPro 2017. $*p<0.05$, $**p<0.01$, $***p<0.001$ (ANOVA, Bonferroni test); n=5 of distinct samples with mean±SEM. G907 used for these experiments was kindly provided by Genentech.

(D) ATPase assays in the presence of MsbA inhibitor G907

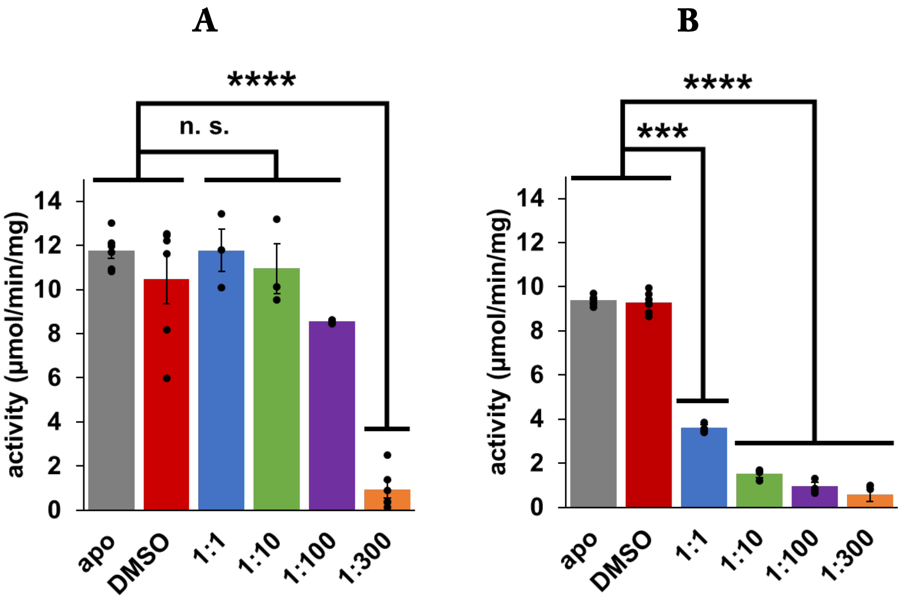

Figure S4: Determining the optimal G907 concentration for MAS-NMR experiments on MsbA proteoliposomes. **(A)** ATPase activity upon titration of G907 to MsbA in DMPC/DMPA liposomes (LPR=75 mole/mole). A large reduction at a stoichiometry of MsbA:G907 of 1:300 is observed. **(B)** ATPase activity upon titration of G907 to MsbA in POPE/POPG liposomes (LPR=75 mole/mole). Here, G907 at a much lower stoichiometry is needed for inhibiting the MsbA activity, starting already MsbA:G907 of 1:1.

The MsbA ATPase activity was determined based on previous studies using MsbA proteoliposomes incubated with or without G907 in assay buffer (*50 mm HEPES, 50 mm NaCl, 5 mM ATP, 10 mm* $MgCl_2$).[4,5] The release of inorganic phosphate was detected at a fixed ATP concentration of 5 mM at OD_{850}. The reaction was stopped by using 12% w/v sodium dodecyl sulfate (SDS) and colored in two consecutive steps, firstly, with a 1:1 mixture of 12% w/v ascorbic acid and 2% w/v ammonium molybdate in 1 M HCl and finally, using a mixture of 2% w/v sodium citrate, 2% w/v sodium meta-arsenite, and 2% v/v acetic acid. G907 used for these experiments was kindly provided by Genentech.

****p<0.001, ****p<0.0001* (ANOVA, Bonferroni test); n=3 of distinct samples with mean±SEM).

(E) Comparison between DMPC/DMPA and POPE/POPG proteoliposomes

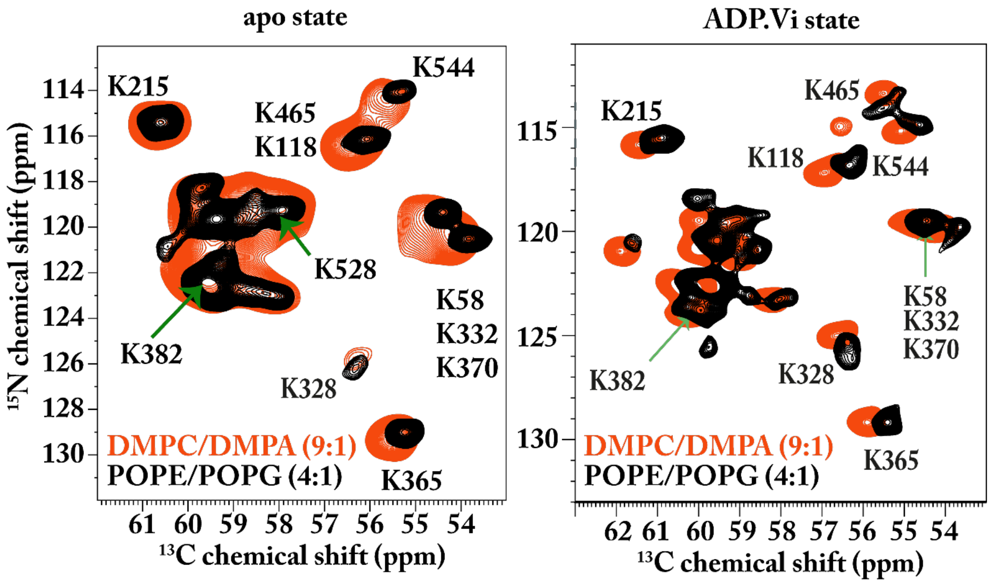

Figure S5: NCA spectra of [^{13}C,^{15}N-K]-MsbA in DMPC/DMPA (red) and POPE/POPG (black). Cross peaks appear sharper in POPE/POPG and some shifts are observed reflecting the differences in membrane properties.

(F) Lipid interactions of G907

Our and published data[6] show that the MsbA inhibitors like G907 reduce cell growth (Fig. S4), which means that they must be able to cross the outer membrane and penetrate the inner membrane. To further illustrate the membrane interaction of G907, ^{1}H-MAS-NOESY experiments on G907 in POPE/POPG lipid bilayers were carried out. POPE/POPG phospholipids are found mainly in the periplasmic leaflet of the asymmetric outer membrane and the symmetric inner membrane of *E. coli* bacteria.[8] ^{1}H resonances of the aromatic regions of G907 are well distinguished from the lipid acyl chains (Fig. S7). From the NOE cross peak intensities of intermolecular lipid - G907 correlations (Fig. S8), a qualitative location of the inhibitor within the bilayer can be derived. With a NOESY mixing time of 50 ms, cross peaks between G907 and the acyl chains of the POPE/POPG lipids are already visible and become much more pronounced at longer mixing times such as 400 ms (Fig. S8). Overall, these interactions are observed in all parts of the lipids, but the most pronounced signals are found in the acyl chains. The drug-lipid interactions are also observed in the lipid head groups but with lower NOESY peak intensities. These data underline the assumption that G907 must be able to penetrate lipid bilayers to reach the MsbA binding site.

A comparison between G907-lipid cross peaks between DMPC and POPE/POPG is shown in Fig. S9. In DMPC, smaller NOEs are observed indicating weaker interactions which is in line with the observation that a higher G907 concentration is needed on DMPC compared to POPE/POPG to reach similar inhibitory effects (Fig. S5).

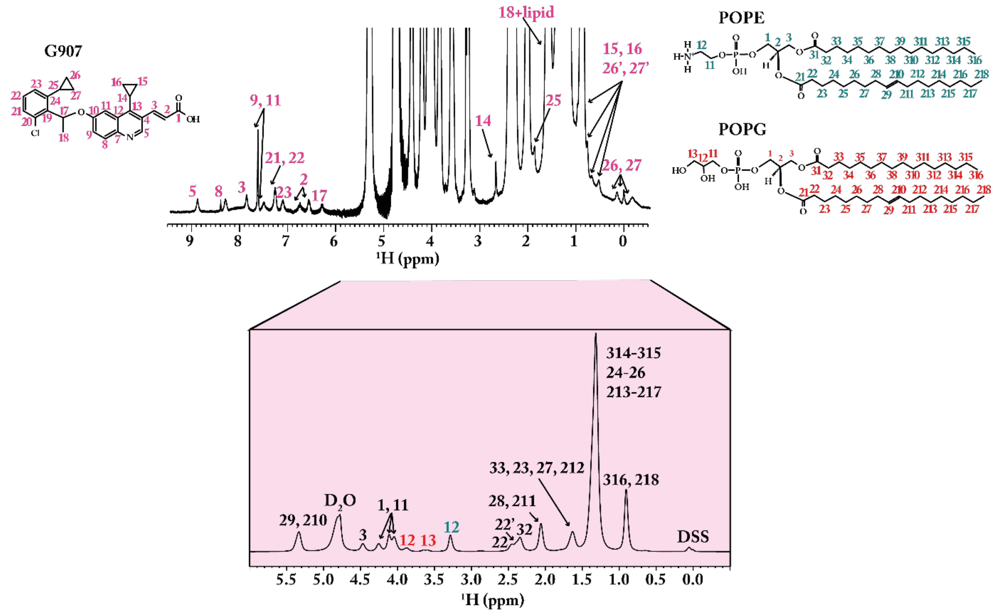

Figure S6: ^{1}H resonance assignments of G907 and POPE/POPG (4:1). One-dimensional proton spectra were recorded at 600 MHz (290 K, 10 kHz MAS). Assignments in black refers to peaks for both POPE and POPG. Assignments in green, red, and pink refer to POPE, POPG, and G907, respectively.

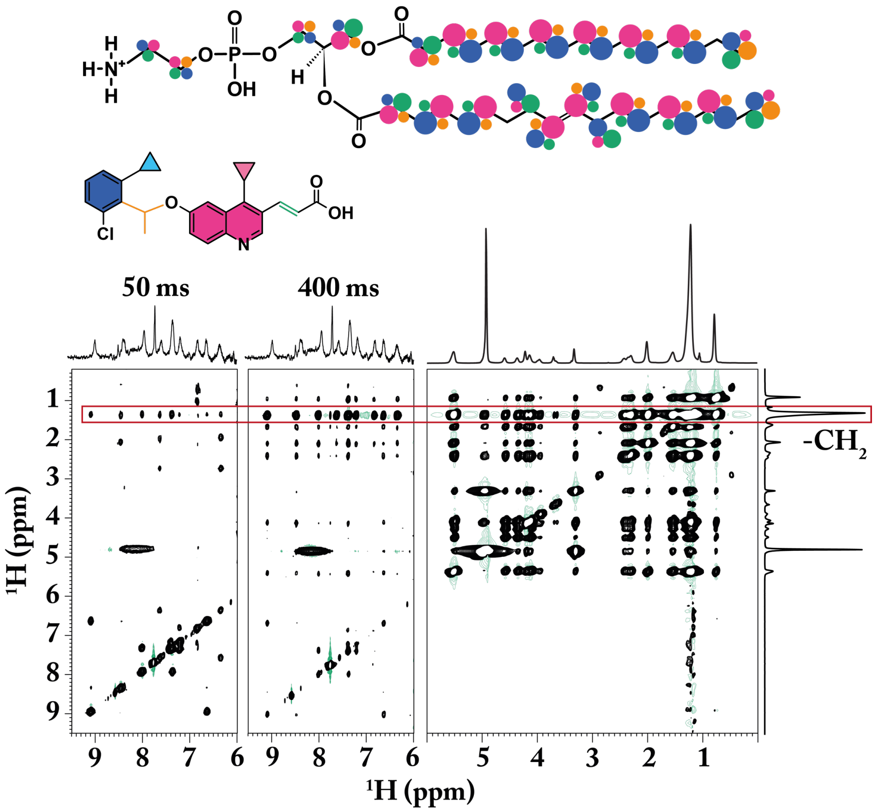

Figure S7: ^{1}H MAS NOESY spectra of POPE/POPG and G907 for a mixing time of 50 and 400 ms (mixing time for overall maximum cross peak intensities). The aromatic signals of G907 occur between 7.5-9.5 ppm and are well separated from the lipid signals (below 6 ppm). Cross-peak intensities of G907 inside the lipid bilayer are indicated with the red box at 1.3 ppm. Colors in the POPE lipid structure indicate cross peak intensities with the G907 drug region indicated in the same colors.

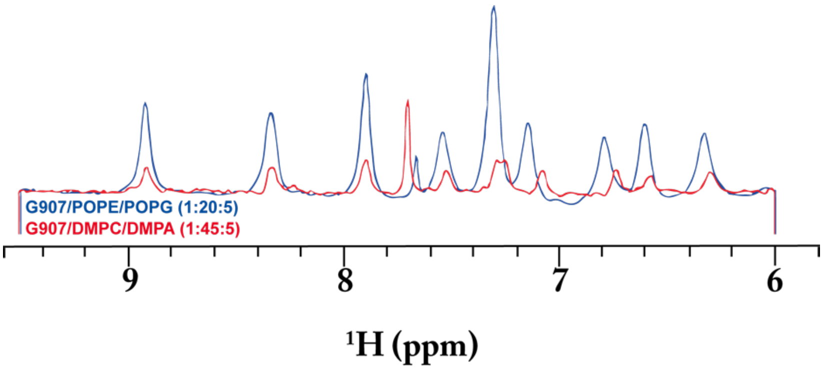

Figure S8: Slice along ω1 (1.2 ppm) of the spectrum in Fig. S8 in comparison with the same slice taken from the spectrum of G907 in DMPC/DMPC lipid bilayers (not shown). The spectra were normalized to the diagonal CH_2 resonance. Both samples contained the same G907:lipid ratio. The difference in NOE peak intensity is 3.4 times (scaling factor = 0.5513) higher for POPE/POPG compared to DMPC/DMPA.

(G) Chemical shifts

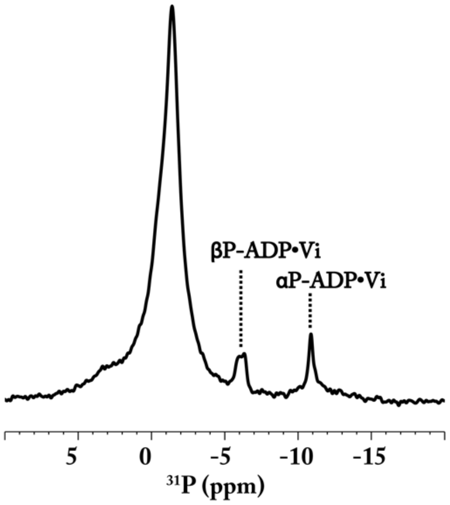

Figure S9: Example for probing the successful trapping of the ADP-Vi bound state of MsbA by ^{31}P CP-MAS NMR (MsbA : DMPC = 1 : 75 mole/mole). Under cross-polarization (CP), ^{31}P nuclei within immobilized parts of the non-frozen sample become visible, such as lipids (signal around 0 ppm) and αP-, βP-ADP.Vi when bound to MsbA. Spectra were recorded as described before [9,10].

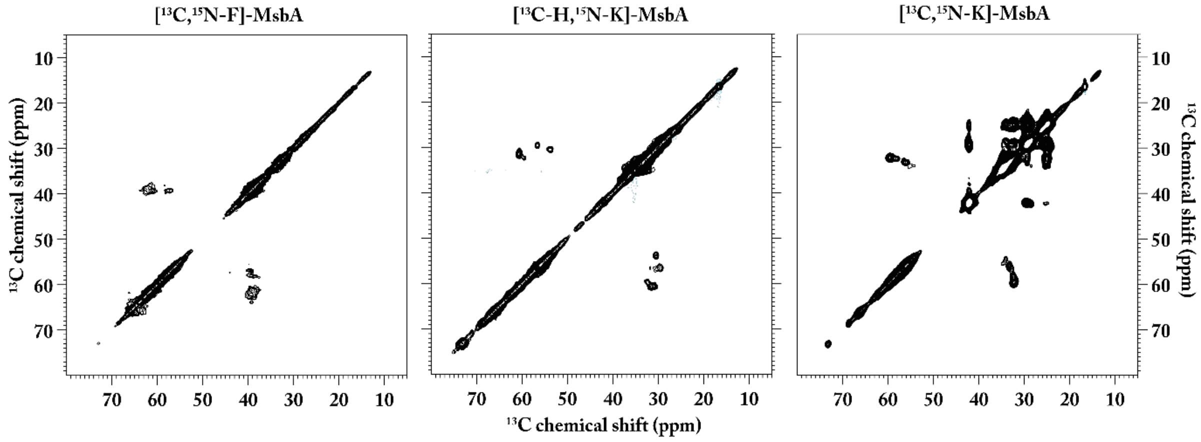

Figure S10: ^{13}C-^{13}C PDSD spectra of [^{13}C, ^{15}N-F]-MsbA (left), [^{13}C-H, ^{15}N-K]-MsbA (middle) and [^{13}C, ^{15}N-K]-MsbA (right). The expected ^{13}C-^{13}C correlations are observed in all three cases demonstrating successful labeling. Spectra were recorded at 600 MHz, 270K with a MAS rate of 11 kHz. A mixing time of 20 ms was used. All spectra were recorded on MsbA reconstituted into DMPC/DMPA lipid bilayers. ^{13}C chemical shift referencing was carried out indirectly to DSS using the C'-resonance of Alanine at 179.85 ppm.

(H) Tables

Table S1: Observed chemical shift changes in CH2 and CH2

Coupling Helix	Nuclei	δ(ADP.Vi) - δ(apo) [ppm]	δ(H33342)- δ(apo) [ppm]	δ(H33342+ ADP.Vi)- δ(ADP.Vi) [ppm]	δ(G907)- δ(apo) [ppm]	NMR spectra and samples
CH1	**F115- C'**	+0.55	+0.05	-0.3	0.33	NCO, [^{13}C,^{15}N-F]-MsbA
	**F116-N**	+1.65	+1.6	-0.04	-1.62	NCO, [^{13}C,^{15}N-F]-MsbA
	**K118-N**	+0.83	+0.09	-0.02	-0.03	NCA, [^{13}C,^{15}N-K]-MsbA
	**K118-Ca**	+0.6	+0.12	-0.04	-0.08	NCA, [^{13}C,^{15}N-K]-MsbA
CH2	**H214-C'**	+0.08	-1.81	0.35	-	NCO, [^{13}C-H,^{15}N-K]-MsbA
	**K215-N**	+0.3	8.01	-0.23	0.0	NCO, [^{13}C-H,^{15}N-K]-MsbA
	**K215X-N**	+0.1	0.38	-0.2	-0.23	NCA, [^{13}C,^{15}N-K]-MsbA
	**K215X-Ca**	+0.8	0.11	-0.3	0.0	NCA, [^{13}C,^{15}N-K]-MsbA

Supplementary References

1 Lyu, J. *et al.* Structural basis for lipid and copper regulation of the ABC transporter MsbA. *Nat Commun* **13**, 7291, doi:10.1038/s41467-022-34905-2 (2022).

2 Mi, W. *et al.* Structural basis of MsbA-mediated lipopolysaccharide transport. *Nature* **549**, 233-237, doi:10.1038/nature23649 (2017).

3 Kehlenbeck, D. M. *et al.* Cryo-EM structure of MsbA in saposin-lipid nanoparticles (Salipro) provides insights into nucleotide coordination. *FEBS J* **289**, 2959-2970, doi:10.1111/febs.16327 (2022).

4 Kaur, H. *et al.* The ABC exporter MsbA probed by solid state NMR - challenges and opportunities. *Biol Chem* **396**, 1135-1149, doi:10.1515/hsz-2015-0119 (2015).

5 Chifflet, S., Torriglia, A., Chiesa, R. & Tolosa, S. A method for the determination of inorganic phosphate in the presence of labile organic phosphate and high concentrations of protein: application to lens ATPases. *Anal Biochem* **168**, 1-4, doi:10.1016/0003-2697(88)90002-4 (1988).

6 Ho, H. *et al.* Structural basis for dual-mode inhibition of the ABC transporter MsbA. *Nature* **557**, 196-201, doi:10.1038/s41586-018-0083-5 (2018).

7 Alexander, M. K. *et al.* Disrupting Gram-Negative Bacterial Outer Membrane Biosynthesis through Inhibition of the Lipopolysaccharide Transporter MsbA. *Antimicrob Agents Chemother* **62**, doi:10.1128/AAC.01142-18 (2018).

8 Wang, J., Ma, W. & Wang, X. Insights into the structure of Escherichia coli outer membrane as the target for engineering microbial cell factories. *Microb Cell Fact* **20**, 73, doi:10.1186/s12934-021-01565-8 (2021).

9 Kaur, H. *et al.* Unexplored Nucleotide Binding Modes for the ABC Exporter MsbA. *J Am Chem Soc* **140**, 14112-14125, doi:10.1021/jacs.8b06739 (2018).

10 Kaur, H. *et al.* Coupled ATPase-adenylate kinase activity in ABC transporters. *Nat Commun* **7**, 13864, doi:10.1038/ncomms13864 (2016).
