# Supplementary material for: Probing the allosteric NBD-TMD crosstalk in the ABC transporter MsbA by solid-state NMR
Source: Commun Biol. 2024 Jan 5;7:43. doi: 10.1038/s42003-023-05617-0 (PMC10770068; doi:10.1038/s42003-023-05617-0)
Supplement: Supplementary file 2 — Description of Additional Supplementary Files [file 42003_2023_5617_MOESM2_ESM.pdf]

Description of Additional Supplementary Files

File name: Supplementary Data 1
Description: Numerical values of predicted chemical shifts shown in Fig. 4.

File name: Supplementary Data 2
Description: Numerical values for the ATPase assays shown in Fig. S2.

File name: Supplementary Data 3
Description: Numerical data for the cell growth assays shown in Fig. S3.

File name: Supplementary Data 4
Description: Numerical values for the ATPase assays shown in Fig. S4.
